# Analysis of KRAS, NRAS, and BRAF Mutations, Microsatellite Instability, and Relevant Prognosis Effects in Patients With Early Colorectal Cancer: A Cohort Study in East Asia

**DOI:** 10.3389/fonc.2022.897548

**Published:** 2022-06-28

**Authors:** Yang Li, Jun Xiao, Tiancheng Zhang, Yanying Zheng, Hailin Jin

**Affiliations:** ^1^ Gastroenterology Endoscopy Center, Affiliated Hospital of Nanjing University of Chinese Medicine (Jiangsu Province Hospital of Chinese Medicine), Nanjing, China; ^2^ Department of Pathology, Affiliated Hospital of Nanjing University of Chinese Medicine (Jiangsu Province Hospital of Chinese Medicine), Nanjing, China

**Keywords:** KRAS, NRAS, BRAF, microsatellite instability, gene mutation, early colorectal cancer

## Abstract

**Background:**

Early colorectal cancer (ECRC) refers to any size of colorectal cancer (CRC) whose depth of invasion is limited to the mucosa and submucosa. About 10% of patients with ECRC die from cancer after surgery. KRAS, NRAS, and BRAF mutations and microsatellite instability (MSI) are considered diagnostic and prognostic markers in CRC. However, their characteristics in ECRC and whether postoperative chemotherapy based on them will benefit ECRC patients or not remain unknown.

**Patients and Methods:**

Patients with ECRC and 298 patients with advanced colorectal cancer (ACRC) were collected in our hospital from January 2013 to December 2015. The Amplification Refractory Mutation System (ARMS)-PCR was used to perform the KRAS, NRAS, and BRAF mutant tests.

**Results:**

In ECRC patients, 43 cases of KRAS mutation were found, accounting for 69.35%. Interestingly, among KRAS mutations, there were 10 KRAS multi-site mutation patients (16.13% in 62 ECRC patients). Moreover, the NRAS mutation rate was 3.23% but no BRAF mutation was found and only 1 case of MSI-High was detected. KRAS mutation was only related to the depth of tumor invasion whereas KRAS multi-site mutations were related to mucus components and tumor size. As far as NRAS is concerned, mutations were associated with elevated CEA, mucus components, and the depth of tumor invasion. Notably, compared with 2.35% KRAS multi-site mutation in ACRC, the rate of KRAS multi-site mutation in ECRC was much higher. Furthermore, Cox regression analysis revealed that KRAS mutation could be an independent prognostic factor of ECRC in patients who have undergone endoscopic resection or surgery.

**Conclusion:**

Patients with ECRC might benefit from KRAS mutation testing but not from postoperative chemotherapy.

## Introduction

Colorectal cancer (CRC) is one of the most aggressive digestive system malignancies, and poses a major threat to human health (1). Early CRC (ECRC) refers to any size of CRC whose depth of invasion is limited to the mucosa and submucosa, regardless of whether there is lymph node metastasis. Simultaneously, high-grade intraepithelial neoplasia is recognized as ECRC. Among them, those limited to the mucosal layer are intramucosal cancers, and those that infiltrate into the submucosa but do not invade the extrinsic muscle layer are submucosal cancers (2). According to the American Joint Committee on Cancer (AJCC) tumor staging manual 8th edition, ECRC refers to CRC stage I (T_1_N_0_M_0_ or T_2_N_0_M_0_) (3). High-quality endoscopic examination and advanced endoscopic resection techniques such as endoscopic submucosal dissection (ESD) performed in experienced centers have increased the survival rates >90% at 5 years for patients with ECRC (4). However, still about 10% of patients with ECRC die from cancer after surgery. How to further improve the survival time of patients with ECRC is being pursued, so that ECRC can be completely cured.

Multistep genetic mutations in oncogenes and DNA repair genes occur over time, resulting in neoplastic progression in CRC. Previous studies have shown that the mutations of proto-oncogenes KRAS, NRAS, and BRAF were keys that have a significant value in CRC (5). When a mutation occurs, proto-oncogenes are continuously activated, and upstream genes cannot regulate downstream genes, which stimulates the proliferation and differentiation of CRC cells. In addition, it has been suggested that microsatellite instability (MSI), which was caused by DNA mismatch repair, plays an important role in the development of CRC (6). Microsatellite consists of repeated sequences of 1–6 nucleotides (7). The distribution characteristics are different from 15 to 65 nucleotides tandem repeats of small satellite DNA, which is mainly located near the ends of chromosomes (8). In line with the frequency of MSI, it can be distinguished into three types: microsatellite stability (MSS), low microsatellite instability (MSI-L), and high microsatellite instability (MSI-H). At present, MSI-L and MSS are considered the same type relative to MSI-H. Genetic studies of CRC have developed rapidly and many significant studies demonstrated that MSI was closely related to CRC (9). It is recommended to combine the detection of RAS and RAF mutations with MSI for the guidance of chemotherapy and prognostic analysis of patients with CRC stage II, III, and IV (5, 10, 11). However, for patients with ECRC, especially those who had undergone ESD treatment, whether genetic testing and additional treatment, including surgery and chemotherapy, are beneficial still needs to be determined.

This study firstly explored the characteristics of KRAS, NRAS, and BRAF mutations and MSI in ECRC patients and looked for mutation-associated factors. Then, the characteristics of KRAS, NRAS, and BRAF mutations and MSI in patients with ECRC were described. Finally, the survival analysis of ECRC patients was performed to provide a basis for ECRC gene mutation detection and postoperative chemotherapy.

## Materials and Methods

### Patients and Data Collection

The cohort included patients with CRC diagnosed clinically and pathologically from January 2013 to December 2015 in our hospital. ECRC referred to CRC stage I (T_1_N_0_M_0_ or T_2_N_0_M_0_) as per the guild of AJCC tumor staging manual 8th edition, while other CRC stages were classified into ACRC. Simultaneously, high-grade intraepithelial neoplasia was recognized as ECRC. Information including the patients’ gender, age, endoscopy and surgery information, pathological characteristics, and survival time was collected. All patients had undergone ESD or surgery to obtain a complete pathology specimen.

The inclusion criteria were as follows (1): a definite diagnosis of CRC under postoperative pathology; (2) complete clinical and medical records, which can assess tumor staging; and (3) complete follow-up to 60 months or death.

The exclusion criteria were as follows: (1) adjuvant chemotherapy, radiotherapy, or targeted drug therapy before operation; (2) colorectal tumors that metastasized from tumors of other organs; (3) anal canal skin tumors; and (4) incomplete clinical data or lost visits.

### Tissue Samples and DNA Extraction

Paraffin specimens of tumor tissues and paired normal tissues were obtained from the Department of Pathology of our hospital. After dewaxing, DNA were extracted using a paraffin DNA extraction kit (Tiangen Biochemical Technology Co., Ltd., Beijing, China) as recorded in the manual. DNA sample quality and concentration assessment was performed using a Nano Drop 2000 spectrophotometer (Thermo Fisher Scientific, Inc., USA). The samples were prepared as 50 ng/μl and stored at −20°C.

### KRAS, NRAS, and BRAF Mutation Test

In this study, the Amplification Refractory Mutation System (ARMS)-PCR was used to perform the KRAS, NRAS, and BRAF mutant tests. The human KRAS and NRAS mutation detection kit (ADx-KN03-MX, AmoyDx Biological Co., Ltd., Xiamen, China) and the human BRAF v600e detection kit (ADx-BR01, AmoyDx Biological Co., Ltd., Xiamen, China) were used to detect the relevant mutation sites. Following the kit instructions, a positive control (PC) and a negative control (NPC) were set up. The reaction system was 65.8 μl of DNA/PC/NPC + 4.2 μl of K and NRAS/BRAF MIX, and the reaction conditions were as follows: (1) 95°C for 5 min; (2) 95°C for 25 s, 64°C for 20 s, 72°C for 20 s, cycle 15 times; and (3) cycle at 93°C for 25 s, 60°C for 35 s, 72°C for 20 s, 31 times. At 60°C in stage (3), signals from hydroxyfluorescein and 5-hexachlorofluorescein phosphoramidate were collected and analyzed. The Ct value of each mutation in the sample reaction tube and the Ct value of the externally controlled reaction tube were determined according to the amplification curve. If Ct ≤ 25, a positive mutation was determined; if 25 < Ct ≤ 30, the test would be performed again. A positive mutation was also determined if Ct ≤ 30 in the repetitive test.

### MSI Test

We used fluorescence multiplex PCR-capillary electrophoresis to measure 5 single nucleotide markers of MSI: Bat26, Bat25, NR-24, CAT25, and MONO-27. The repeat sites’ 5 single-nucleotide markers are as follows: (A)26, (T)25, (T)24, (T)25, and (A)27. MSI was tested using the human MSI detection kit (AmoyDx Biological Co., Ltd., Xiamen, China). PCR amplification was performed firstly, and the reaction system was MIX1 7 μl + MIX2 10 μl + DNA extraction solution 17 μl. The reaction conditions were set as follows: (1) 95°C for 5 min; (2) 95°C for 25 s, 62°C for 40 s, 65°C for 30 s, 10 times; (3) 93°C for 25 s, 58°C for 40 s, 65°C for 30 s, 21 times; and (4) 56°C for 10 min. Finally, capillary electrophoresis was performed for 45 min, and the amplified products were detected. The MSI status was determined using Gene Mapper version 4.1 (Thermo Fisher Scientific, Inc., USA). The fluorescence signal of tumor was observed and recorded compared with normal tissue. Among the five marks, if two or more marks changed, it was judged to be MSI-H, and if there was one mark change, it was judged to be MSI-L. If there was no change in the five markers, it is determined as MSS.

### Survival Analysis

All enrolled patients were followed up as outpatients regularly, and relevant information was recorded. If the patients died or were still alive at 60 months, the follow-up was terminated. The survival period was recorded in months (maximum 60 months), and if the number of extra days was 15 days or more, it is counted as 1 month. Postoperative chemotherapy referred to the use of platinum chemotherapeutics and complete 6 treatment courses of chemotherapy. Kaplan–Meier analysis was performed using R software version 3.6 and related factors were screened. Survival curves were plotted for positive results. COX analysis was also performed using R software to clarify the relationship between factors and survival.

### Statistical Analysis

SPSS (version 22.0; IBM Corp., Armonk, NY, USA) was used for data compilation and statistics. All data were expressed as mean ± SD. Logistic regression analysis was used to identify the clinical and pathological associations of KRAS, NRAS, and BRAF mutations and MSI in patients with ECRC to find mutation-related factors. The general information; KRAS, NRAS, and BRAF mutations; and MSI of ECRC and ACRC were compared by chi-square test, and patient age was compared by ANOVA test. The characteristics of KRAS and NRAS mutation sites were compared by chi-square test and Fisher’s exact test. However, if the amount of data in one group was more than 4 times that of the other group, no statistical analysis was performed, but only a description. Survival analysis was performed using Kaplan–Meier test and COX test by R software. *p* < 0.05 was considered to indicate a statistically significant difference.

## Results

### General Information and Clinical Characteristics of KRAS, NRAS, and BRAF Mutations and MSI in ECRC

This cohort study included 360 consecutive patients registered with an initial diagnosis of CRC in our hospital within a 3-year period from January 2013 to December 2015. Among the included patients, there were 140 male patients and 220 female patients with a median age of 61.89 ± 11.43 years. Also, in these 360 CRC patients, 62 patients were diagnosed with ECRC while the remaining 298 patients were diagnosed with ACRC. Of the 62 patients diagnosed with ECRC, 37 were male and 25 were female. Their mean age was 61.85 ± 10.31, of whom 32 patients were 65 years or older.

Through the ARMS-PCR method, 43 cases of KRAS mutation were found, accounting for 69.35% of ECRC patients. After analyzing the rate of every mutation site, it was found that KRAS exon2 G12S/D was the most mutated site with a total of 21 cases (**Table 1**). After KRAS exon2 G12S/D, there were 13, 11, 8, and 1 patient with KRAS exon2 G13D, KRAS exon4 K117N/A146T/V/P, KRAS exon2 G12C/R/V/A/G13C, and KRAS exon3 A59T/Q61K mutations, respectively. No mutation was detected at the KRAS exon3 Q61L/R/H site. Of particular interest to us is that among KRAS mutations, there were 10 KRAS multi-site mutations, accounting for 16.13% of ECRC patients. At the same time, ARMS-PCR testing also found 2 cases of NRAS mutation and the mutation rate was 3.23%. NRAS mutation occurred on the NRAS exon2 G13D site. In 62 patients with ECRC, fluorescence multiplex PCR-capillary electrophoresis found 1 case (1.61%) of MSI-H and 2 cases of MSI-L (3.23%).

**Table 1:** KRAS, NRAS, and BRAF mutation sites and rates in patients with ECRC.

Gene	Test no.	Mutation no.	Mutation rate	Test section	Mutation symbol	DNA base change	Mutation no.	Mutation rate
KRAS	62	43	69.35%	exon2	G12S	34G>A	21	48.84%
					G12D	35G>A		
				exon2	G12C	34G>T	8	18.60%
					G12R	34G>C		
					G12V	35G>T		
					G12A	35G>C		
					G13C	37G>T		
				exon2	G13D	38G>A	13	30.23%
				exon3	A59T	175G>A	1	2.33%
					Q61K	181C>A		
				exon3	Q61L	182A>T	0	0
					Q61R	182A>G		
					Q61H	183A>C		
					Q61H	183A>T		
				exon4	K117N	351A>C	11	25.58%
					K117N	351A>T		
					A146T	436G>A		
					A146V	437C>T		
					A146P	436G>C		
NRAS	62	2	3.23%	exon2	G12D	35G>A	0	0
					G12S	34G>A		
				exon2	G13D	38G>A	2	100%
				exon2	G13R	37G>T	0	0
					G12C	34G>T		
					G12V	35G>T		
					G12A	35G>C		
					G13V	38G>T		
				exon3	Q61R	182A>G	0	0
					Q61K	181C>A		
					Q61L	182A>T		
					Q61H	183A>C		
				exon4	A146T	436G>A	0	0
BRAF	62	0	0	exon15	V600E	600V>E	0	-

### Clinicopathological Association of KRAS, NRAS, and BRAF mutations and MSI

The above findings showed that KRAS, NRAS, and BRAF mutations and MSI had their unique features in patients with ECRC. In order to further explore the possible factors, we used logistic regression analysis to study the clinicopathological relationship of KRAS, NRAS, and BRAF mutations and MSI in ECRC patients. We collected and analyzed factors such as gender, age, tumor site, tumor size, presence or absence of mucus components, lymph node metastasis, invasion depth, tumor budding, vascular invasion, pathological type, and clinical manifestations. KRAS mutation was related to the depth of tumor invasion and clinical manifestations. KRAS multi-site mutation was related to the presence or absence of mucus components and tumor size. NRAS mutation was associated with elevated CEA, the presence or absence of mucus in the tumor, the depth of tumor invasion, and clinical manifestations. The number of MSI-H cases was small, and no relevant factors had been found in the study (**Table 2**).

**Table 2:** Clinicopathological association of KRAS, NRAS, and BRAF mutation and MSI in ECRC.

Variables	No.	KRAS mutation (*n* = 43)		KRAS multi-sites mutation (*n* = 10)		NRAS mutation (*n* = 2)		MSI (MSI-H = 1)	
		MT	*p*	Y	*p*	MT	*p*	MSI-H	*p*
**Gender**			0.452		0.924		0.511		0.403
Male	37	27		7		2		0	
Female	25	16		3		0		1	
**Age**			0.879		0.552		1.000		1.000
<65	35	24		7		1		1	
≥65	27	19		3		1		0	
**CEA≥5**			0.926		0.804		0.029		0.177
Yes	11	8		2		2		1	
No	51	35		8		0		0	
**Tumor location**			0.065		0.388		0.658		0.592
Rectum	30	28		7		1		1	
Left hemi-colon	15	11		2		0		0	
Right hemi-colon	17	4		1		1		0	
**Tumor size**			0.678		0.035		0.560		0.747
≤1 cm	5	3		3		0		0	
1< *n ≤*3	39	24		7		2		1	
>3	18	16		0		0		0	
**Mucoid carcinoma**			0.325		<0.01		0.019		1.000
Yes	9	8		7		2		0	
No	53	35		3		0		1	
**Lymphatic metastasis**			0.758		1.000		1.000		1.000
Yes	4	3		0		0		0	
No	58	40		10		2		1	
**Invasive depth**			0.001		0.296		0.024		1.000
M1–SM1	52	41		10		0		1	
SM2–SM3	10	2		0		2		0	
**Tumor budding**			0.966		0.653		0.918		0.958
Grade 1	58	40		10		2		1	
Grade 2	3	2		0		0		0	
Grade 3	2	1		0		0		0	
**Vascular invasion**			1.000		0.065		1.000		1.000
Yes	3	2		1		0		0	
No	59	41		9		2		1	
**Pathological type**			1.000		0.296		0.024		1.000
Adenocarcinoma	59	42		10		2		1	
Others	3	2		0		0		0	
**Clinical manifestations**			0.024		–		–		1.000
Obstruction	1	1		0		0		0	
Blood in stool	10	7		0		0		1	

### Percentage of ECRC and ACRC in KRAS/NRAS/BRAF Mutations and MSI

Through this study, it was found that the KRAS, NRAS, and BRAF mutation rates in all included cases were 47.50%, 4.72%, and 3.89%, and MSI-H accounted for 6.39% of the total. The KRAS mutation rate in ECRC was 69.4% and that in ACRC was 42.5%. At the same time, compared with 2.35% KRAS multi-site mutation in ACRC, the rate of KRAS multi-site mutation in ECRC was 16.1%. The percentage of NRAS and BRAF mutations and MSI-H of the two groups of patients is presented in **Table 3**. The mutation site rates of KRAS exon2 G12S/D, G13D, KRAS exon4 K117N/A146T/V/P, and NRAS exon2 G13D were high in ECRC patients (**Table 4**).

**Table 3:** Differences in KRAS/NRAS/BRAF mutation and MSI between ECRC and ACRC.

Variables	ECRC	Rate (%)	ACRC	Rate (%)
**Gender**				
Male	37	59.7	103	34.6
Female	25	40.3	195	65.4
**Age (years)**	61.85 ± 10.31		61.91 ± 11.51	
**KRAS**				
Wild type	19	30.6	170	57.5
Mutant type	43	69.4	128	42.5
**KRAS multi-site mutation**				
No	52	83.9	291	97.7
Yes	10	16.1	7	2.3
**NRAS**				
Wild type	60	96.8	283	95.0
Mutant type	2	3.2	15	5.0
**BRAF**				
Wild type	62	100	284	95.3
Mutant type	0	0	14	4.7
**MSI**				
MSI-H	1	1.6	22	7.4
MSI-L/MSS	61	98.4	276	92.6

**Table 4:** Mutant sites in early and advanced cancer.

No.	Test section	Mutation symbol	ECRC (*n* = 62)		ACRC (*n* = 298)	
			Mutation No.	Mutation Rate	Mutation No.	Mutation Rate
1	KRAS exon2	G12S/D	21	33.87%	59	19.8%
2	KRAS exon2	G12C/R/V/A/G13C	8	12.9%	34	11.41%
3	KRAS exon2	G13D	13	20.97%	27	9.06%
4	KRAS exon3	A59T/Q61K	1	1.62%	2	0.67%
5	KRAS exon3	Q61L/R/H	0	0	6	2.01%
6	KRAS exon4	K117N/A146T/V/P	11	11.74%	6	2.01%
7	NRAS exon2	G12D/S	0	0	1	0.33%
8	NRAS exon2	G13D	2	3.24%	2	0.67%
9	NRAS exon2	G13R/V/G12C/V/A	0	0	5	1.68%
10	NRAS exon3	Q61R/K/L/H	0	0	9	3.02%
11	NRAS exon4	A146T	0	0	0	0

### Survival Analysis of ECRC

**Figure 1:**
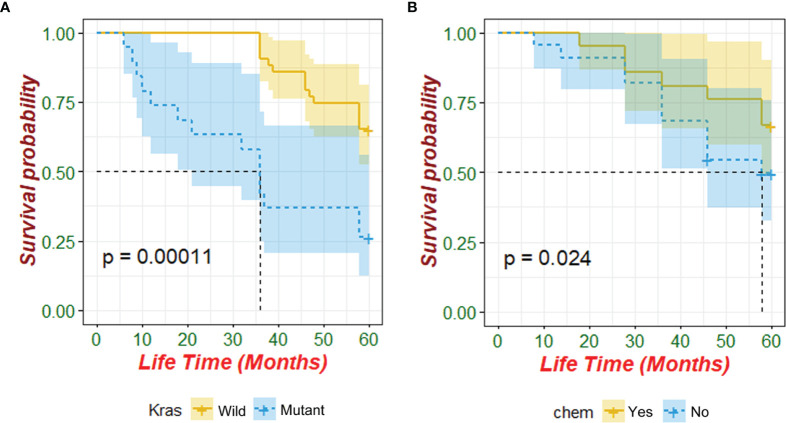
Prognostic value of KRAS mutation and postoperative chemotherapy for patients with ECRC (chem: postoperative chemotherapy).

Kaplan–Meier analysis identified that patients with wild-type KRAS and postoperative chemotherapy had significantly better overall survival (*p*<0.05, **Table 5** and **Figure 1**). Multivariate Cox regression analysis revealed that only wild-type KRAS (HR = 0.441, 95% CI 0.195–0.993, *p* = 0.048) was the independent prognostic factor of ECRC in patients (**Table 5**).

**Table 5:** Survival analysis of ECRC.

Variables	Case Number	Overall Survival (Months)	*p*	Hazard Ratio	95% Confidence Interval	*p*
**Gender**			0.623			0.277
Male	37	49.40 ± 2.96				
Female	25	48.87 ± 2.86				
**Age**			0.130			0.897
≥65 years	32	46.72 ± 3.14				
<65 years	30	51.60 ± 2.62				
**KRAS**			<0.01	0.441	0.195–0.993	0.048
Wild type	19	55.02 ± 1.31				
Mutant type	43	35.63 ± 4.86				
**KRAS multi-site mutation**			0.092			0.762
No	52	55.50 ± 3.01				
Yes	10	47.85 ± 2.36				
**NRAS**			0.233			0.108
Wild type	60	49.12 ± 2.12				
Mutant type	2	48.00 ± 10.00				
**BRAF**			–			–
Wild type	62	–				
Mutant type	0	–				
**MSI**			0.14			0.105
MSI-H	1	36 ± 0				
MSI-L/MSS	61	49.30 ± 2.10				
**Post-chemotherapy**			0.024			0.735
Yes	29	56.38 ± 1.46				
No	33	42.67 ± 3.30				

Post-chemotherapy, postoperative chemotherapy.

## Discussion

The occurrence and development of CRC are a complex process involving multi-gene changes and multi-stage accumulation. A large amount of evidence suggested that the occurrence and development of CRC are related to the disorder of the regulatory mechanism of the RAS-RAF-MAPK signaling pathway. Under normal circumstances, when the transmembrane cell receptor EGFR is activated by an extracellular ligand, the conformation of the RAS protein changes, and it becomes active RAS-GTP, which activates the intracellular RAS-RAF-MAPK signaling pathway and affects the gene change inside the cell. This signaling pathway controls gene transcription of cells, which affects multiple processes such as cell proliferation, differentiation, and apoptosis. Previous studies have shown that proto-oncogenes RAS and RAF are key molecules upstream of the RAS-RAF-MAPK signaling pathway in patients with CRC. When a mutation occurs in the early stage of CRC, the RAS-RAF-MAPK pathway is continuously activated, so that RAS and RAF are not regulated, and intestinal epithelial cells have irreversibly turned the mode of tumor change to cancerous. Therefore, RAS and RAF mutations are regarded as CRC promoter genes and a sign of ECRC.

KRAS is located on human chromosome 12 and encodes a 21-kDa RAS protein that contains two subtypes, namely, KRAS4A and KRAS4B (12). Previous studies have shown that the mutation rate of KRAS in patients with CRC is 37%–56%, and the mutation status is related to tumor size, tumor location, degree of tumor differentiation, lymph node metastasis, and other factors (13–16). Sideris (17) et al. showed that KRAS mutation status may affect the prognosis of ECRC, as this is linked to distant recurrence. However, it is confusing that the relationship of KRAS with staging of CRC is not exactly the same (18). KRAS mutation occurs mostly at the N-terminal codons 12, 13, and 61, and the codon 12 mutation is the most common (19). In this study, the mutation rate of KRAS in all patients was 47.5%, which was consistent with previous reports. In patients with ECRC, the KRAS mutation rate was 69.35%. KRAS exon2 G12S/D, KRAS exon2 G13D, and KRAS exon4 K117N/A146T/V/P were the most significantly susceptible site, and the mutation was only related to the longitudinal infiltration depth of ECRC. What was interesting to us was that this study also found that the KRAS multi-site mutation rate in ECRC was 16.13%, and it was 2.35% in ACRC. In addition, the KRAS multi-site mutation rate was related to the presence or absence of mucus components and tumor size. Thus, the KRAS mutation rate and multi-site mutation might be meaningful in ECRC. It is inferred that KRAS mutation or KRAS multi-site mutation may be one of the characteristics of ECRC. KRAS mutation is related to the depth of tumor invasion and the prognosis of ECRC. In ECRC, the proportion of KRAS mutations is high, but the 5-year survival rate of patients increased. This is inconsistent with many known studies that show that KRAS mutations are the hallmark of poor prognosis for CRC (19, 20). Perhaps in the process of CR evolution, there are some unknown factors that affect the KRAS mutation. The specific mechanism in this process still requires us to continue in-depth research.

NRAS is a subtype of RAS family, located on the short arm of chromosome 1 (1p22-p32), and also encodes RAS protein. Compared with KRAS, the mutation rate of NRAS in CRC patients is relatively low, about 1.2% to 4.2% (20–22). This study found that the NRAS mutation rate in patients with ECRC is 3.23%, which was similar to that of advanced cancer. The whole mutation rate of NRAS in patients with CRC was consistent with previous reports. All NRAS mutation in ECRC occurred at the NRAS exon2 G13D, while ACRC mutation sites were NRAS exon3 Q61R/K/L/H, NRAS exon2 G13R/V/G12C/V/A, NRAS exon2 G13D, and NRAS exon2 G12D/S in descending order. Furthermore, the increase of CEA, the presence or absence of mucus components, the longitudinal invasion depth, and clinical manifestations of ECRC were related to NRAS mutation. However, due to the small number of NRAS mutation cases of ECRC in this study, further relevant factors might need to be determined.

BRAF is a downstream gene of RAS in the RAS-RAF-MAPK signaling pathway, and its mutation is homogeneous. Almost all BRAF mutation occurs at the V600E site in exon 15. The mutation rate of BRAF in CRC patients was about 10%, and it is associated with poor prognosis (23, 24). Previous studies confirmed that Asians have a relatively low mutation rate of BRAF compared to Westerners. Won et al. (25) reported that the mutation rate is about 4% in South Korea. Previous studies also suggested that BRAF and KRAS mutations were independent of each other and would not occur at the same time. However, this science conception has been updated now. Gong et al. (26) retrospectively analyzed the gene sequences of 138 metastatic CRCs. They found that RAS and RAF had a mutation at the same time, and the incidence rate was 1.4%. In this study, the BRAF mutation rate was 4.7% in patients with ACRC, and there was no BRAF mutation in patients with ECRC. A 57-year-old woman with ACRC had both KRAS and BRAF mutations in this study. As a result, the significance of BRAF in the diagnosis and treatment of ECRC needs further exploration.

MSI is closely related to various tumors especially CRC. With the implementation of the human genome project, scientists sought to detect the relevant methods. Four methods, namely, next-generation sequencing (NGS), fluorescent multiplex PCR and CE, immunohistochemistry (IHC), and single-molecule molecular inversion probes (smMIPs), have been used to detect MSI. Among these four methods, fluorescent multiplex PCR and CE is regarded as the gold standard, which can help the MSI accuracy rate reach 100% through the detection of five 5 MS sites: BAT-26, NR-21, BAT-25, MONO-27, and NR-24 genes (27). Fujiyoshi et al. (28) found that the prognosis of MSI-H CRC was good, while the prognosis of MSS and MSI-L CRC was poor. Another study suggested that MSI-H has a good prognosis, a high 5-year survival rate, and a low recurrence rate and deterioration rate in patients with stage I and II CRC, but patients with stage III CRC had the opposite results (29). In this study, there was 1 case of MSI-H and 2 cases of MSI-L, accounting for 1.61% and 3.23% of patients with ECRC, respectively. The only MSH-H case was a 57-year-old female patient with rectal cancer. The diameter of the rectal cancer was 2.8 × 2.5 cm, with the depth of invasion into the mucosal layer, and there is no lymph node metastasis or distant metastasis. The only thing worth noting is that she once had hematochezia. A study from South Korea indicated that the proportion of MSI-H was 7.2%, which was lower than that of European and American countries (25). Additionally, the proportion of MSI-H in this study was only 1.61%, lower than the data of South Korea, suggesting that the proportion of MSI-H in CRC patients in East Asian countries is indeed low. The difference of the MSI-H percentages between Western and East Asian countries may be related to the following factors: Firstly, there are differences in ethnicity and gene polymorphisms between East Asians and Westerners, leading to certain differences in the gene repeated sequences of MSI-H (30). Secondly, the difference is also related to the differences in the clinicopathological classification between Eastern and Western patients. MSI-H may be related to the depth of tumor invasion, tumor location, lymph node metastasis, and pathological type, which are different in Eurasians (31, 32). Finally, the difference may be related to the detection site. There are two types of MSI detection sites: the NCI Panel with 2 single nucleotides (BAT-25 and BAT-26) + 3 double nucleotides (D5S346, D2S123, and D17S250) sites and the Pentaplex Panel with 5 single nucleotides (Bat26, Bat25, NR-24, CAT25, and MONO-27) sites. Recent studies indicated that the NCI Panel seemed to be more suitable for East Asians (33) although the NCI Panel and the Pentaplex Panel had good consistency. However, this view has not been recognized widely. Due to the small number of cases of MSI-H in ECRC patients, further relevant research containing more patients should be implemented in order to draw more reliable conclusions.

The guidelines in Japan and the United States both recommend preoperative or postoperative chemotherapy for patients with stage II, III, and IV CRC, while chemotherapy for patients with ECRC is not explicitly recommended or prohibited (11, 34, 35). This cohort study further analyzed the survival of patients with ECRC after analyzing the characteristics of KRAS, NRAS, and BRAF mutations and MSI. This analysis provided evidence for addressing the need for chemotherapy in ECRC patients after endoscopy treatment or surgery. The current study had demonstrated that KRAS mutation could affect patient survival and be an independent factor in determining the prognosis of ECRC. Furthermore, for ERCR patients with KRAS mutations, indications for additional surgery after ESD can be explored. However, postoperative chemotherapy might affect ECRC patient survival, while it was not an independent factor for the prognosis of ECRC. Therefore, postoperative chemotherapy may not be recommended for patients with ECRC.

This study has several limitations. To begin with, it was only a single-center cohort study. Multicenter and prospective studies that included a larger sample need to be conducted to further increase the credibility and level of evidence. In addition, this study did not explore the relationship of KRAS, NRAS, and BRAF mutations and MSI with the recurrence and metastasis of ECRC, which may become the direction of our future research.

Above all, patients with ECRC have a high rate of KRAS mutation and KRAS multi-site mutation. More importantly, KRAS mutations are associated with the survival of patients with ECRC. Patients with ECRC will benefit from KRAS mutation testing but not from postoperative chemotherapy. For ERCR patients with wild-type KRAS, additional surgery or other therapy methods after ESD can be explored. It is hoped that RAS and RAF could become objective indicators for clinical decision-making regarding whether additional treatment such as surgery and chemotherapy is required for patients after ESD. Furthermore, the status of KRAS may guide the diagnosis and prognosis of ERCR patients to some extent. New methods targeting KRAS such as targeted therapy and traditional Chinese medicine treatment may need to be explored to find a cure for ECRC.

## Data Availability Statement

The original contributions presented in the study are included in the article/supplementary material. Further inquiries can be directed to the corresponding author.

## Ethics Statement

The studies involving human participants were reviewed and approved by the Ethics Committee of the Affiliated Hospital of Nanjing University of Chinese Medicine. The patients/participants provided their written informed consent to participate in this study.

## Author Contributions

YL wrote this manuscript. YL, TZ, and YZ contributed to the mutation test work. HJ assisted with analysis of clinical information. JX made substantial contributions to the design of the study and data analysis. All authors contributed to the article and approved the submitted version.

## Funding

The present study was supported by the Jiangsu Province Science and Technology Innovation and Achievement Transformation Project (grant no. BL2012071), an innovation fund of the Jiangsu Province Hospital of Chinese Medicine (grant no. Y2020CX38 and Y2018CX57), Science and Technology Development Special Project of Jiangsu Provincial Administration of Traditional Chinese Medicine (grant no. 2020zx07) and the Jiangsu Province “333 Project” Training Fund (grant no. BRA2017551).

## Conflict of Interest

The authors declare that the research was conducted in the absence of any commercial or financial relationships that could be construed as a potential conflict of interest

## Publisher’s Note

All claims expressed in this article are solely those of the authors and do not necessarily represent those of their affiliated organizations, or those of the publisher, the editors and the reviewers. Any product that may be evaluated in this article, or claim that may be made by its manufacturer, is not guaranteed or endorsed by the publisher.
